# Intraspecific leaf morphometric diversity in the endangered and narrow endemic *Quercus trojana* subsp. *euboica* (Fagaceae): a quantitative dataset from three natural populations in Euboea, Greece

**DOI:** 10.3897/BDJ.14.e199459

**Published:** 2026-09-29

**Authors:** Fani Lyrou, Nikolaos Tourvas, Filippos Aravanopoulos

**Affiliations:** 1 Laboratory of Forest Genetics, Aristotle University of Thessaloniki, Thessaloniki, Greece Laboratory of Forest Genetics, Aristotle University of Thessaloniki Thessaloniki Greece

**Keywords:** conservation priority, foliar traits, phenotypic plasticity, phenotypic variation, post-fire monitoring

## Abstract

This study provides the first quantitative dataset on leaf morphometric diversity for Quercus trojana subsp. euboica (Papaioannou) K.I.Chr., a narrow endemic oak of Greece. This subspecies is of high conservation priority, listed in the Red Data Book of Rare and Threatened Plants of Greece and its restricted range in Northern Euboea was heavily impacted by a catastrophic wildfire in 2021. We sampled 301 leaves from three natural populations (Kerasia1, Kerasia2 and Prokopi) and processed them using a custom Python pipeline to extract 14 size and shape variables. Statistical analyses (PCA and LDA) showed that, while leaf shape remains relatively stable across populations, there is significant variation in leaf size. Specifically, the Prokopi population, where only understorey individuals were found, had much larger leaves (mean leaf area 1,015 mm^2^) than those in Kerasia1 (648 mm^2^) and Kerasia2 (715 mm^2^). These data establish a baseline for monitoring the species' recovery and will be used alongside future genomic data to better understand the role of phenotypic plasticity in its conservation.

## Introduction

Quercus trojana subsp. euboica (Papaioannou) K.I.Chr. (Fagaceae), is the only endemic oak in Greece, restricted to the ophiolitic (serpentine) substrates of Northern Euboea Island at elevations ranging from near sea level to approximately 600 m a.s.l. in an area of about 3 km^2^ (Papaioannou 1949, Trigas and Iatrou 2006, Trigas et al. 2008, Doganos 2010, Kartsonas and Papafotiou 2010). This taxon is protected under Presidential Decree 67/81 and listed in the Red Data Book of Rare and Threatened Plants of Greece (Phitos et al. 2009). A molecular phylogeographic study of *Quercus* section *Cerris* demonstrated that Q. trojana subsp. euboica shares no nuclear DNA variants with *Q. trojana* Webb and this taxon was recommended to be treated as an independent species (Simeone et al. 2018), as originally described (Papaioannou 1949). However, in the present study, we retain the subspecies rank to ensure consistency with the Greek Red Data Book and the national legal protection acts (e.g. Presidential Decree 67/81), pending a widely adopted formal taxonomic revision. Furthermore, Q. trojana subsp. euboica differs from Q. trojana subsp. trojana in its shrubby habitus, leathery leaves and the white tomentum of the abaxial leaf surface, composed of stellate trichomes and is characterised by its strict association with serpentine substrates (Papaioannou 1949, Simeone et al. 2018).

Despite its conservation importance, Q. trojana subsp. euboica remains amongst the least studied endemic oaks in Europe and pertinent literature is very limited (Papaioannou 1949, Mucina and Dimopoulos 2000, Trigas and Iatrou 2006, Kartsonas and Papafotiou 2010). Owing to this highly fragmented distribution of numerous small, scattered groups, an accurate estimate of the total population size is difficult to obtain. Prior to the 2021 fires, the population was estimated at approximately 10,000 individuals occupying a total area of less than 3 km^2^ (Phitos et al. 2009, Doganos 2010). To date, no comprehensive post-fire demographic assessment has been carried out across the entire distribution range and the number of surviving individuals, therefore, remains unquantified. The species has nonetheless shown considerable historical resilience to fire: following the destruction of its main distribution area by a 1977 forest fire, it recovered successfully through sprouting, without a marked reduction in its occupied range (Phitos et al. 2009). This gap is especially critical following the catastrophic wildfire of August 2021, which burned approximately 51,200 ha in Northern Euboea and affected the largest part of the known range of Q. trojana subsp. euboica. It is noted that we have nevertheless observed that many burnt individuals have resprouted from the stem.

Leaf morphometrics provide a well-established, cost-effective approach for quantifying intraspecific variation in woody perennial species, with demonstrated utility in taxonomic and population differentiation and conservation studies (Neophytou et al. 2007, Viscosi et al. 2009, Aravanopoulos 2010, Di Pietro et al. 2016, Li et al. 2021, Fortini et al. 2025). Herein we present the first quantitative morphometric dataset for Q. trojana subsp. euboica from three natural populations, based on 14 leaf size and shape variables, assessed through descriptive statistics and multivariate analyses, to establish baseline morphometric diversity for this critically endangered and narrow endemic species.

## Materials and Methods

**Field Sampling and Leaf Collection**

Sampling campaigns were conducted in July 2025 across the known distribution range of Q. trojana subsp. euboica in Northern Euboea (Fig. 1 and Fig. 2), targeting all regions with documented occurrence records (Papaioannou 1949, Mucina and Dimopoulos 2000, Doganos 2010). Leaf material was collected from 31 georeferenced individuals originating from three natural populations (sample size in parentheses): Kerasia1 (n = 14), Kerasia2 (n = 11) and Prokopi (n = 6), maintaining a minimum individual distance of 20-30 m to minimise the probability of sampling related individuals. Each individual was georeferenced using GPS coordinates recorded during fieldwork. From each individual, approximately 10 mature, fully expanded, sun-exposed, south-facing leaves free from herbivory damage or pathological symptoms, were sampled from the upper part of the canopy. Leaves were immediately placed in paper tea bags and pressed to minimise deformation during transport. Drying was carried out under ambient conditions through consecutive filter paper changes until leaves reached stable dry weight. The geographic distribution of sampled populations was visualised using a topographic map of Northern Euboea, produced in RStudio (v.2024.12.1+563, Posit Software, Boston, MA, USA), using the leaflet package (Cheng et al. 2024), with an ESRI World Topographic Map as the basemap.

**Image acquisition and leaf morphology measurement**

Dry leaves were scanned under controlled conditions at 600 dpi in colour using a flat surface scanner, providing high-resolution images suitable for morphometric analysis. Approximately 10 leaves were arranged per photograph (30 photographs total, ~ 300 leaves). A millimetric paper was included in each image to provide a spatial reference for scale calibration.

**Scale calibration**

Prior to morphological extraction, each image was calibrated semi-automatically using a custom Python pipeline (Python v.3.12.x, Python Software Foundation, Wilmington DE, USA); incorporating the NumPy (Harris et al. 2020), OpenCV (Bradski 2000), scikit-image (van der Walt et al. 2014) and Matplotlib (Hunter 2007) libraries. For each image, an operator identified two points on the millimetric paper using an interactive Matplotlib-based GUI window and entered the corresponding real-world distance in millimetres (mm). The pixel-to-millimetre conversion factor (px/mm) was computed as the Euclidean pixel distance between the two points divided by the known physical distance. Calibration factors were stored in a JSON file and treated as a fixed, manually reviewed dataset for further analysis.

**Leaf segmentation**

Each image was loaded and converted from colour to greyscale. Background separation was performed via Otsu’s method (Otsu 1979), which determines an optimal threshold by minimising the intra-class variance between foreground (leaf) and background pixels. The resulting binary mask was refined using morphological closing with a rectangular structuring element (15 x 15 px) to bridge shadow-induced gaps at leaf margins. This was followed by morphological opening (5 x 5 px) to remove small noise artefacts. Leaf contours were extracted using a topological structural analysis algorithm cv2.findContours (Suzuki and Abe 1985), specifically retrieving only the outermost boundaries (RETR_EXTERNAL). Finally, objects with an area outside a plausible range (500-50,000 mm^2^) were discarded to exclude non-target elements, such as ruler fragments, debris and image artefacts.

**Morphological measurements**

For each detected leaf contour, a binary mask was generated and processed using the scikit-image library (van der Walt et al. 2014). Morphological descriptors were extracted via the regionprops function and subsequently converted into physical units, based on the specific pixel-to-millimetre (px/mm) calibration factor of each image (Fig. 3, Table 1).

Quality control overlays were generated for each photograph, showing detected contours colour-coded by leaf identity and labelled with their assigned leaf ID, allowing manual inspection prior to downstream analysis. All measurements were exported to a single CSV file for statistical analysis in RStudio environment (v.2024.12.1+563).

**Data analysis**

All statistical analyses were performed in RStudio environment (v.2024.12.1+563). Descriptive statistics were calculated for all 14 morphometric variables per population. Results are presented as box plots generated using the ggplot2 package (Wickham 2016), displaying the distribution of each variable across the three populations: Kerasia1, Kerasia2 and Prokopi.

To explore the multivariate structure of leaf morphometric variation, a Principal Component Analysis (PCA) was performed on the full set of variables after standardisation using the prcomp function in RStudio. A biplot was produced to visualise individual scores and variable loadings, highlighting the axes driving variation. Additionally, a Linear Discriminant Analysis (LDA) was conducted using the MASS package in RStudio (Venables and Ripley 2002) to assess the discriminatory power of the morphometric traits and quantify population-level differentiation.

## Results

A total of 301 leaves were measured across the three populations, Kerasia1 (Fig. 4), Kerasia2 (Fig. 5) and Prokopi (Fig. 6). Quality control overlays confirm successful segmentation of individual leaves across scanned samples. Quality control overlays confirm successful segmentation by visually demonstrating that the automated pipeline accurately traced the outermost boundaries of each leaf, successfully separated the leaf area from the background and assigned unique colour-coded IDs without capturing image artefacts (Figs 4, 5, 6).

**Descriptive statistics**

Marked differences in leaf size were observed amongst populations, with Prokopi consistently exhibiting the largest leaves. Mean leaf area in Prokopi (1,015.2 mm^2^) was approximately 1.5× greater than in Kerasia1 (648.1 mm^2^) and Kerasia2 (715.0 mm^2^). Similar patterns were observed for perimeter, major and minor axis length, Feret diameters and convex area. In contrast, shape-related parameters exhibited low variation amongst populations, indicating that overall leaf shape is more conserved than leaf size. The petiole-to-lamina ratio showed high intraspecific variability within all three populations, reflecting considerable individual-level variation in petiole length relative to lamina length (Fig. 7).

**Multivariate statistics**

The PCA explained more than 75% of the total morphometric variance in low multi-dimensional space, in particular 50.2% (PC1) and 25.1% (PC2) of the total variance, respectively (Fig. 8). PC1 was defined by size-related variables, with convex area, leaf area, perimeter, minor and major axis and Feret diameters having higher loadings, reflecting an overall leaf size gradient across individuals. PC2 exhibited shape variation, with width-to-length ratio, solidity and aspect ratio contributing most, reflecting variation in differential leaf expansion and morphological roundness. In the ordination space, the Prokopi population was clearly separated from Kerasia1 and Kerasia2 populations along PC1, consistent with its reportedly larger leaf size. Kerasia1 and Kerasia2 overlapped along both axes, though they showed differentiation along PC2. The LDA confirmed population-level structuring, with LD1 separating Prokopi from both Kerasia populations and LD2 exhibiting partial discrimination between Kerasia1 and Kerasia2, indicating that, despite their geographical proximity, the two sub-populations maintain distinct phenotypic signatures across several leaf traits (Fig. 9).

## Discussion

This study provides the first quantitative characterisation of intraspecific leaf morphometric diversity in Q. trojana subsp. euboica. Leaf morphometrics constitute one of the most widely applied tools for studying variation in the genus *Quercus*, where both traditional and geometric morphometric approaches have successfully discriminated species and hybrids amongst European oaks (Viscosi et al. 2009). At the intraspecific level, studies across diverse oak lineages have shown that leaf morphology integrates genetic, geographic and climatic signals (Viscosi et al. 2009, Li et al. 2021) and morphological classification remains an important tool for screening species status in oak population genetics (Salvini et al. 2009). Our dataset, therefore, adds a previously missing reference point for a taxon in which quantitative phenotypic information was entirely absent.

The clear separation of Prokopi from the two Kerasia populations in multivariate space, driven by size-related traits, is consistent with the known influence of genetic differentiation due to isolation by distance and of local environment on leaf variation in Quercus (Viscosi et al. 2009, Li et al. 2021, Fortini et al. 2025). Mean leaf area in Prokopi (1,015.2 mm^2^) was approximately 1.5 times greater than in Kerasia1 (648.1 mm^2^) and Kerasia2 (715.0 mm^2^). A comparable magnitude of amongst-population differentiation in leaf traits has been reported in common-garden experiments of other oaks: in *Q. petraea*, mean specific leaf area ranged from 9.95 to 12.39 m^2^ kg^-1^ across nine European provenances grown under uniform conditions, with significant genetic differentiation, associated with the climate of origin (Torres-Ruiz et al. 2019). However, latitudinal gradients in absolute leaf size in oaks seem to reflect phenotypic plasticity rather than genetic differentiation (Torres-Ruiz et al. 2019). As the leaves of the present study were collected in a single season from natural populations, the relative contributions of plastic responses to micro-environmental heterogeneity and of genetic differentiation to the large Prokopi leaf size, cannot yet be separated.

In contrast to leaf size, shape-related variables (solidity, aspect ratio, circularity) exhibited low variation amongst populations, indicating that overall leaf shape is more conserved. This pattern agrees with findings in other *Quercus* species, where leaf shape traits offer reliable diagnostic characters at the species level, whereas size responds more strongly to environmental conditions (Viscosi et al. 2009). The phenotypic consistency of shape indicators in Q. trojana subsp. euboica agrees with the taxonomic distinctness of the taxon established through molecular phylogenetic evidence (Simeone et al. 2018), although a direct morphometric comparison with *Q. trojana* would be needed to test this more thoroughly.

Partial discrimination between Kerasia1 and Kerasia2 further demonstrates that multivariate morphometrics can resolve fine-scale population structure even within the same general local range. Comparable small-scale differentiation occurs in other oaks: leaf morphology is used to check species status of sampled individuals in population-genetic studies of European oaks (Alberto et al. 2010) and, in *Q. aquifolioides*, geographic factors were shown to shape leaf morphology jointly with genetic and climatic drivers (Li et al. 2021). The differentiation between the two geographically proximate Kerasia sub-populations suggests that micro-geographic environmental filters or demographic processes, such as post-fire regeneration structure, may leave measurable morphometric signatures, a hypothesis that can be directly tested with the genomic data currently being generated for this species.

High within-population variability, particularly in the petiole-to-lamina ratio, may reflect micro-environmental heterogeneity on the ophiolitic substrates of Northern Euboea (Trigas and Iatrou 2006). Such intraspecific trait variation is increasingly recognised as a key component of adaptive capacity in oaks where broad ecological amplitude and phenotypic plasticity are highlighted as sources of short-term resilience to climate-driven stress (Ülker and Tavşanoğlu 2026). For a narrow endemic with a fragmented distribution, this standing variation may be critical for post-fire recovery.

From a conservation perspective, the studied morphometric differentiation supports the conservation of each population as a distinct unit. The severely reduced density at Prokopi suggests a potential further range shrinkage following the 2021 megafire and ongoing anthropogenic pressures. The morphometric baseline established herein provides a quantitative reference for tracking future population-level changes along with genomic monitoring tools.

## Figures and Tables

**Figure 1.**
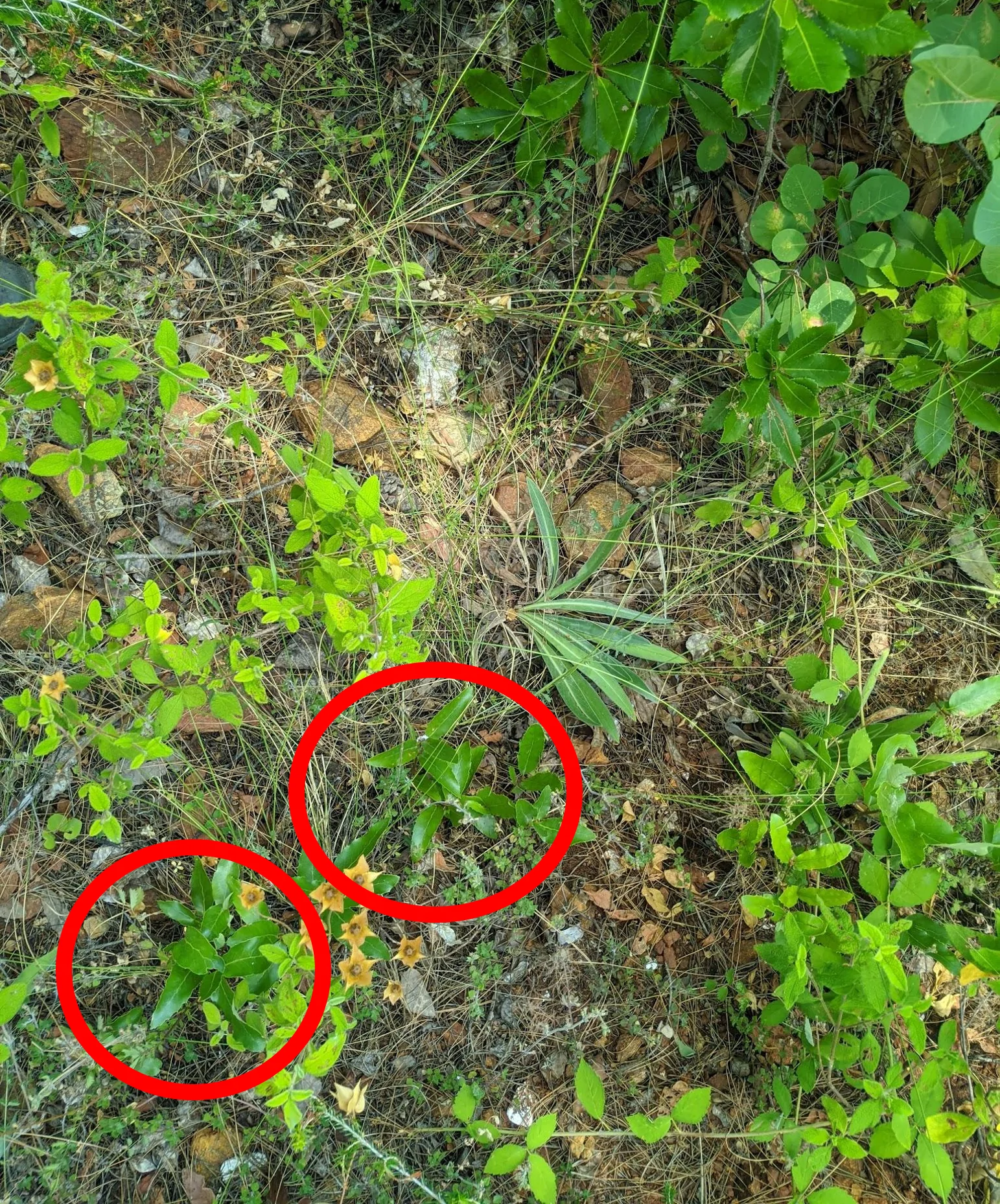
Representative individuals and habitat of Q. trojana subsp. euboica in Northern Euboea, Greece. The photograph presents the typical shrubby growth form and leaf morphology of this taxon in its natural environment.

**Figure 2.**
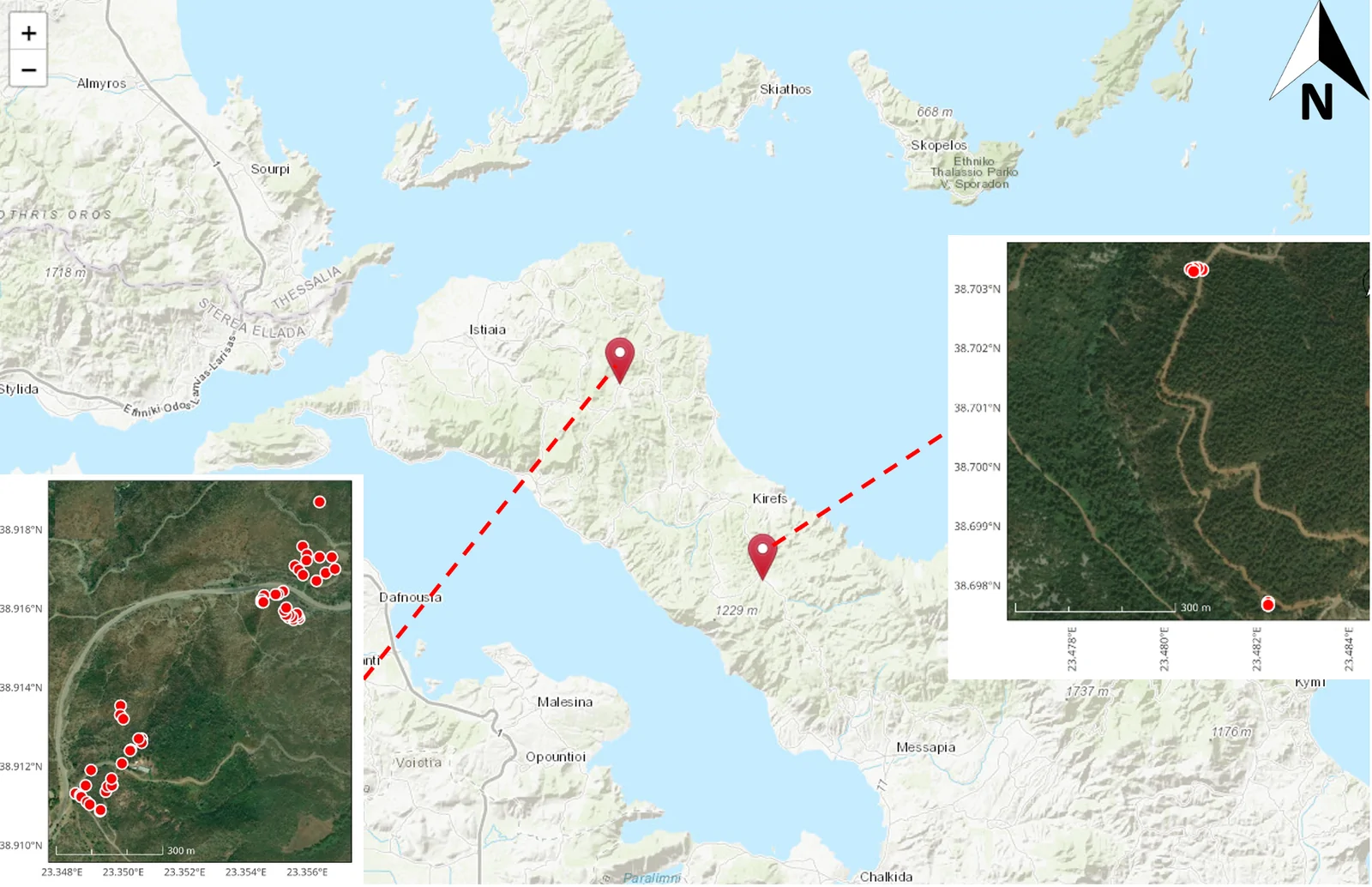
Geographic distribution of Q. trojana subsp. euboica in Northern Euboea, Greece. Red pins indicate sampling sites; insets provide high-resolution satellite views of the specific sub-populations, highlighting the georeferenced locations of individuals within each sampling site.

**Figure 3.**
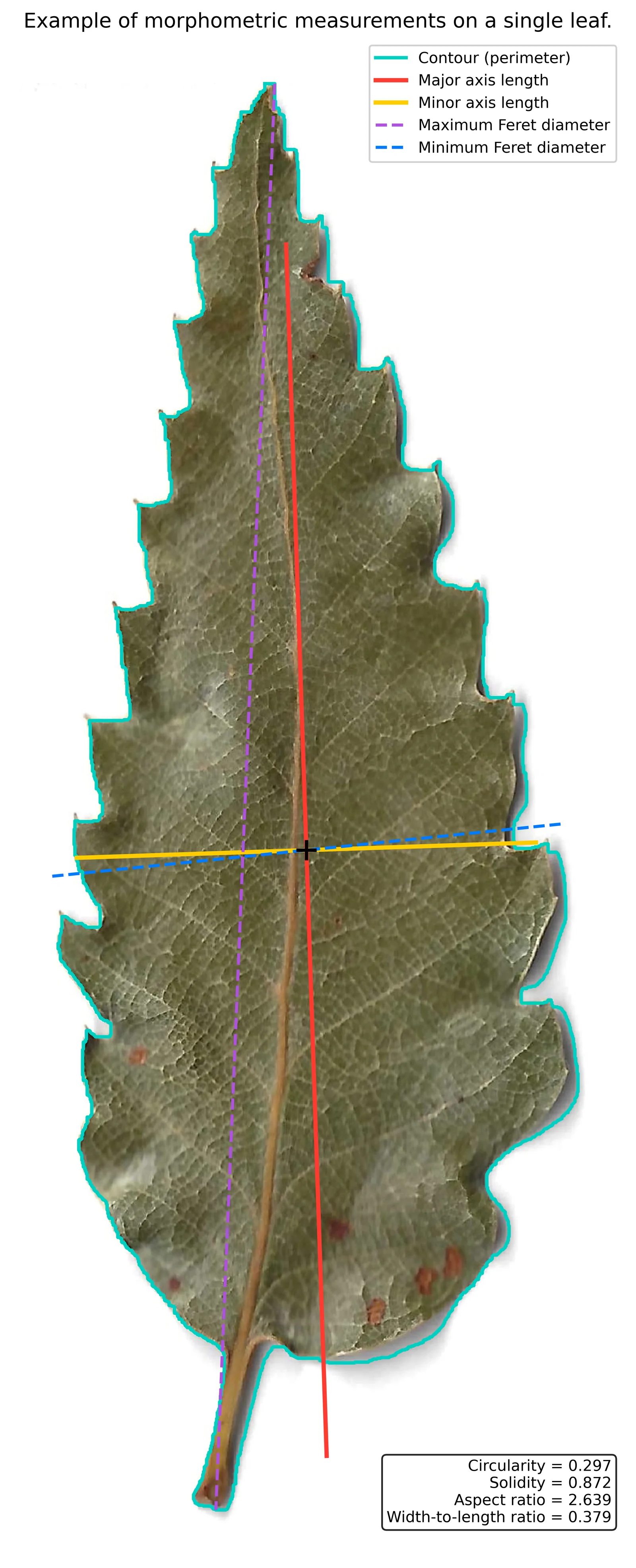
Example of morphometric measurements performed on a single leaf of Q. trojana subsp. euboica. The contour corresponds to the leaf perimeter; solid lines indicate the major and minor axis lengths; dashed lines indicate the maximum and minimum Feret diameters. Derived shape indices (circularity, solidity, aspect ratio, width-to-length ratio) calculated for the same leaf are also reported.

**Figure 4.**
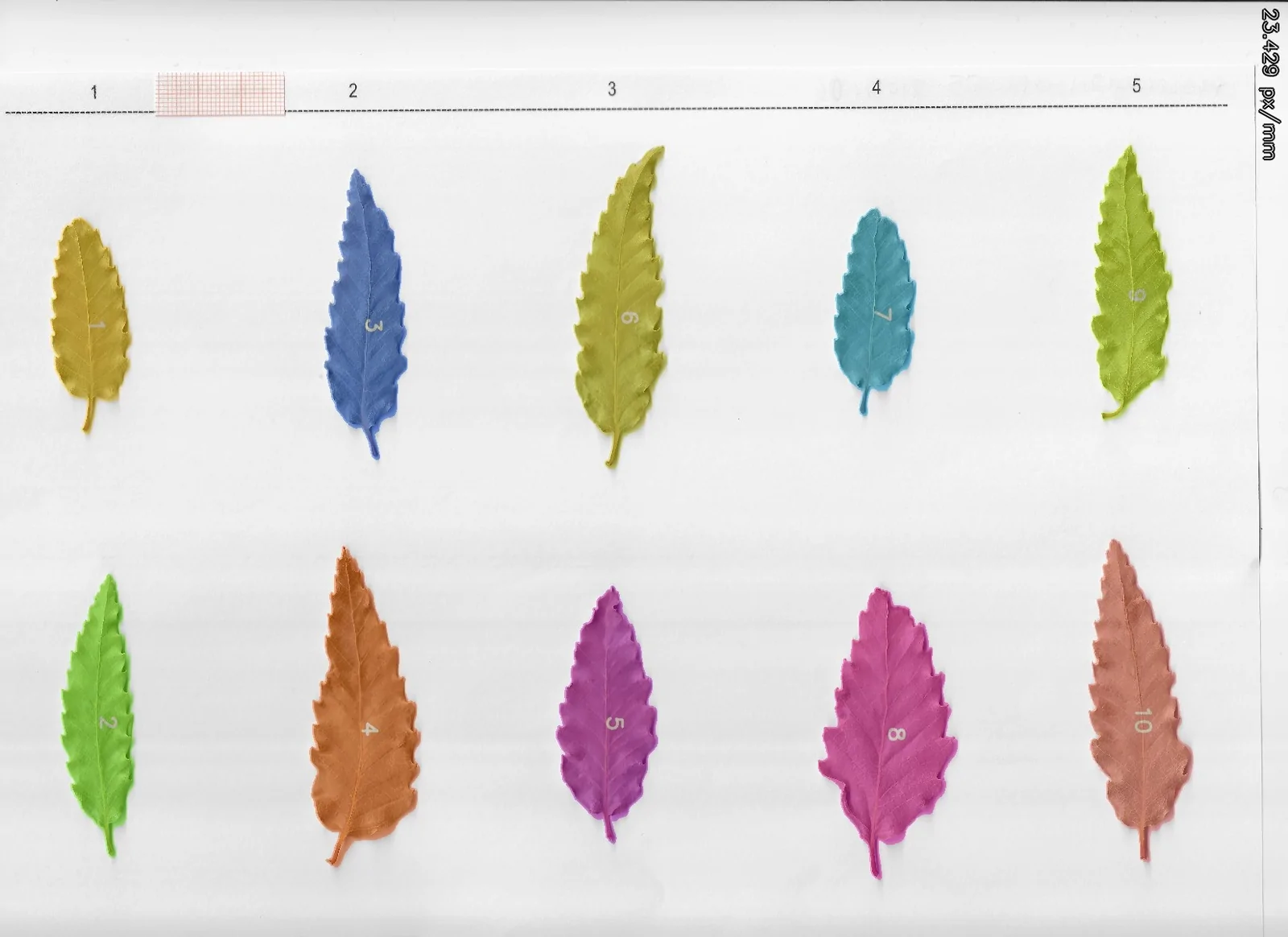
Quality control overlay of scanned leaf images from population Kerasia1 of Q. trojana subsp. euboica. Each leaf contour is colour-coded by individual leaf identity and labelled with its assigned leafID. The dashed line indicates the ruler for scale calibration (23.429 px/mm).

**Figure 5.**
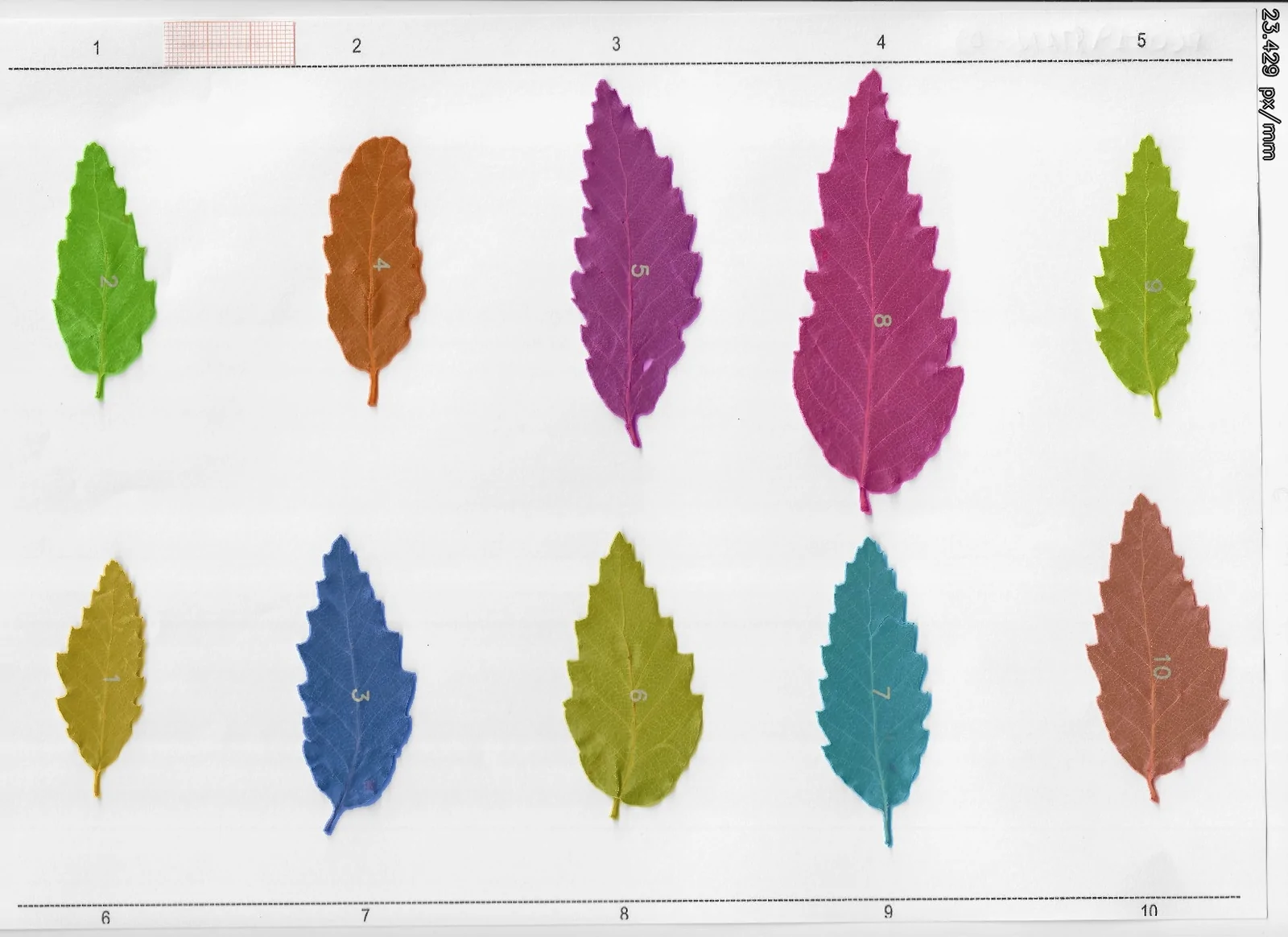
Quality control overlay of scanned leaf images from population Kerasia2 of Q. trojana subsp. euboica. Each leaf contour is colour-coded by individual leaf identity and labelled with its assigned leafID. The dashed line indicates the ruler for scale calibration (23.429 px/mm).

**Figure 6.**
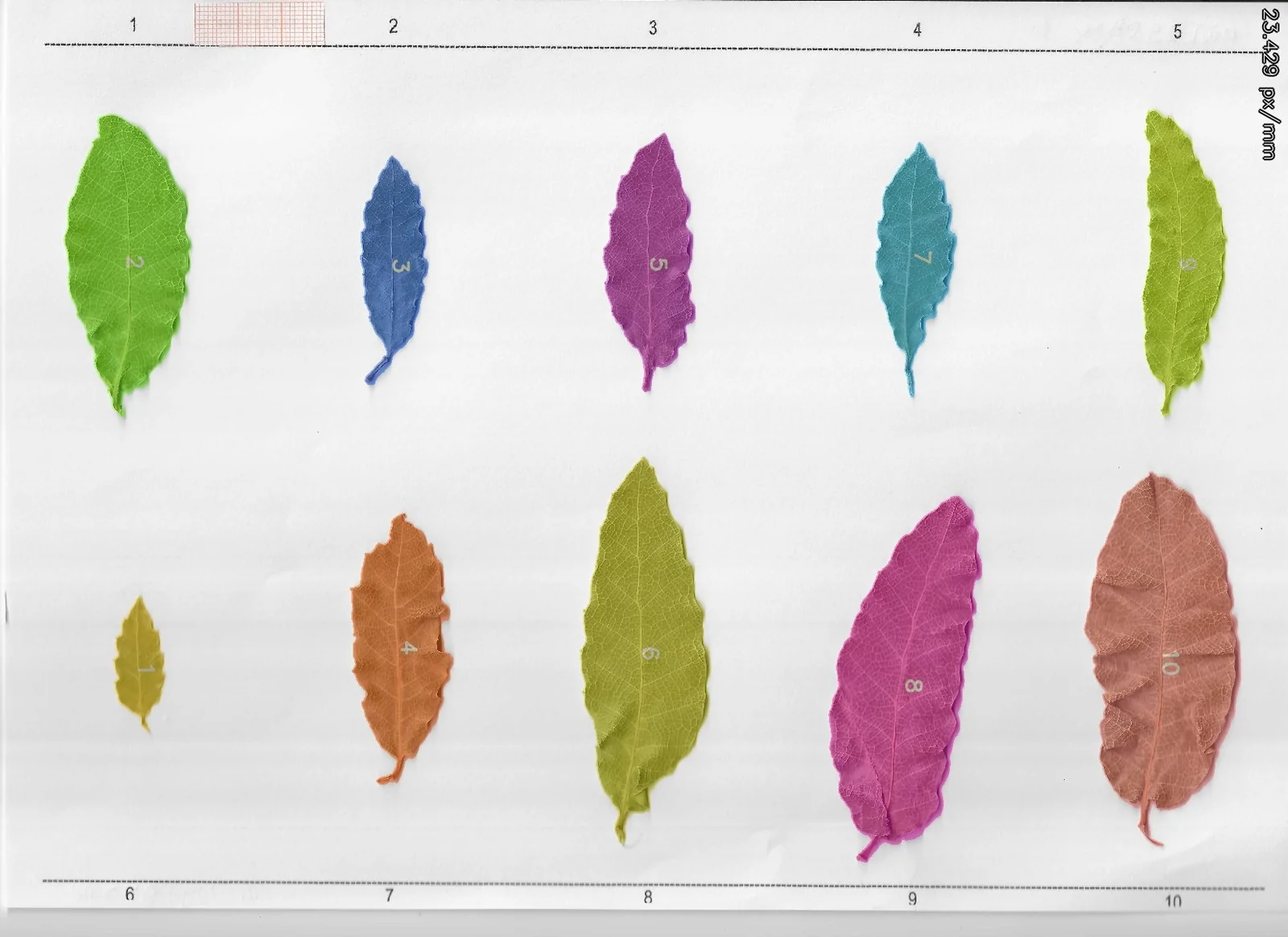
Quality control overlay of scanned leaf images from population Prokopi of Q. trojana subsp. euboica. Each leaf contour is colour-coded by individual leaf identity and labelled with its assigned leafID. The dashed line indicates the ruler for scale calibration (23.429 px/mm).

**Figure 7.**
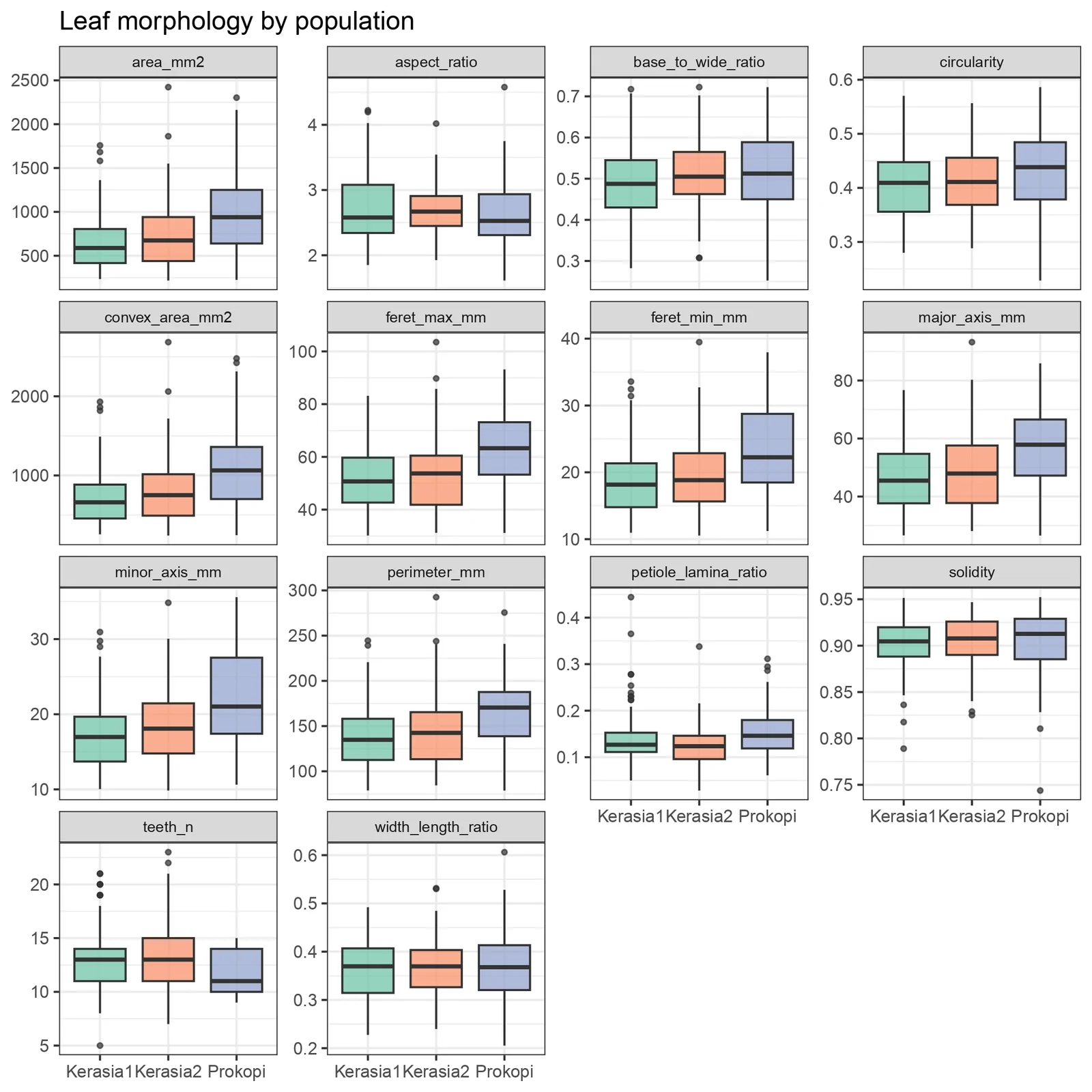
Box plots of the 14 leaf morphometric variables across populations.

**Figure 8.**
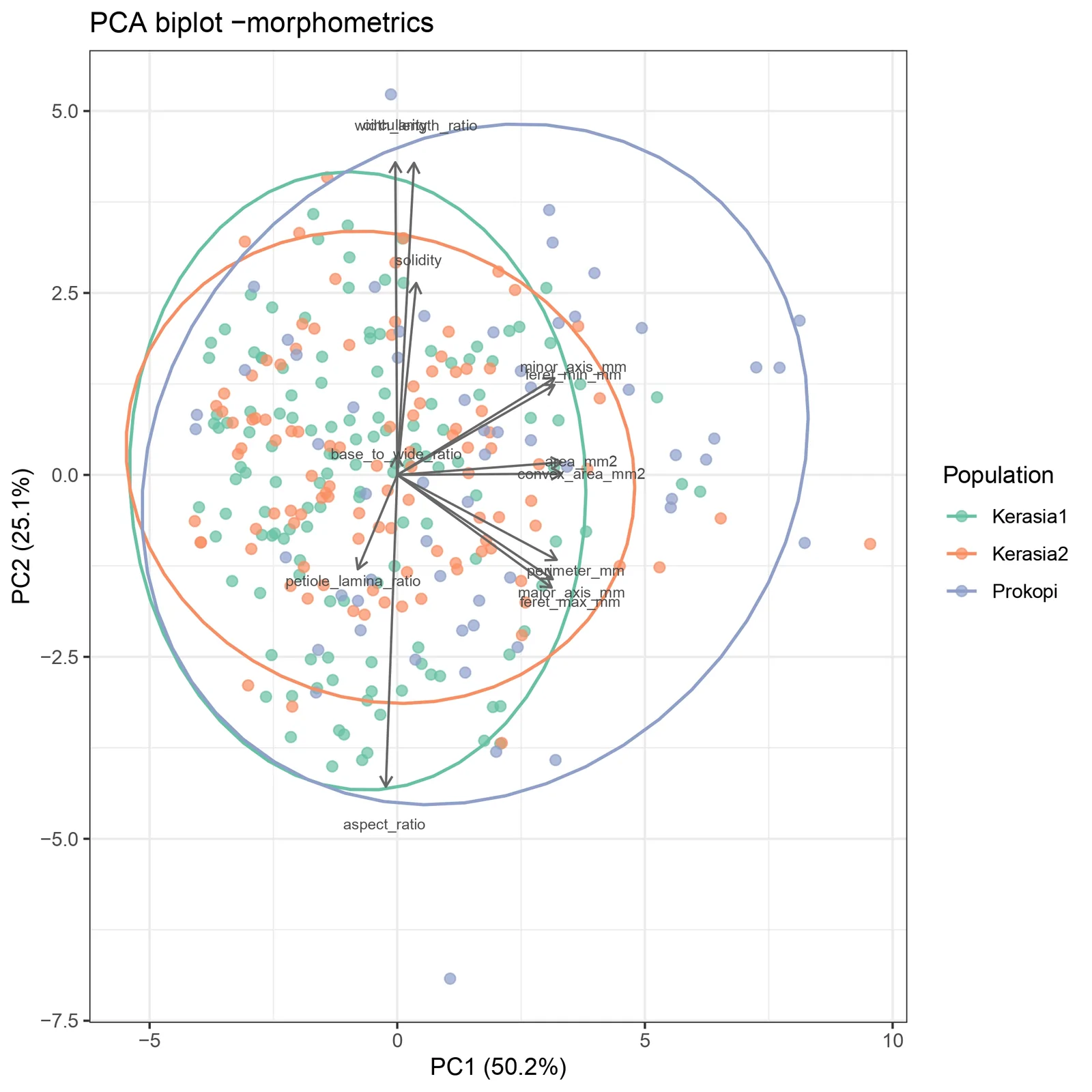
Principal Component Analysis (PCA) biplot of leaf morphometric variation. The first two components account for 75.3% of the total variance (PC1: 50.2%, PC2: 25.1%). Vectors indicate the direction and strength of variable loadings, with PC1 primarily representing a size gradient and PC2 capturing shape variation.

**Figure 9.**
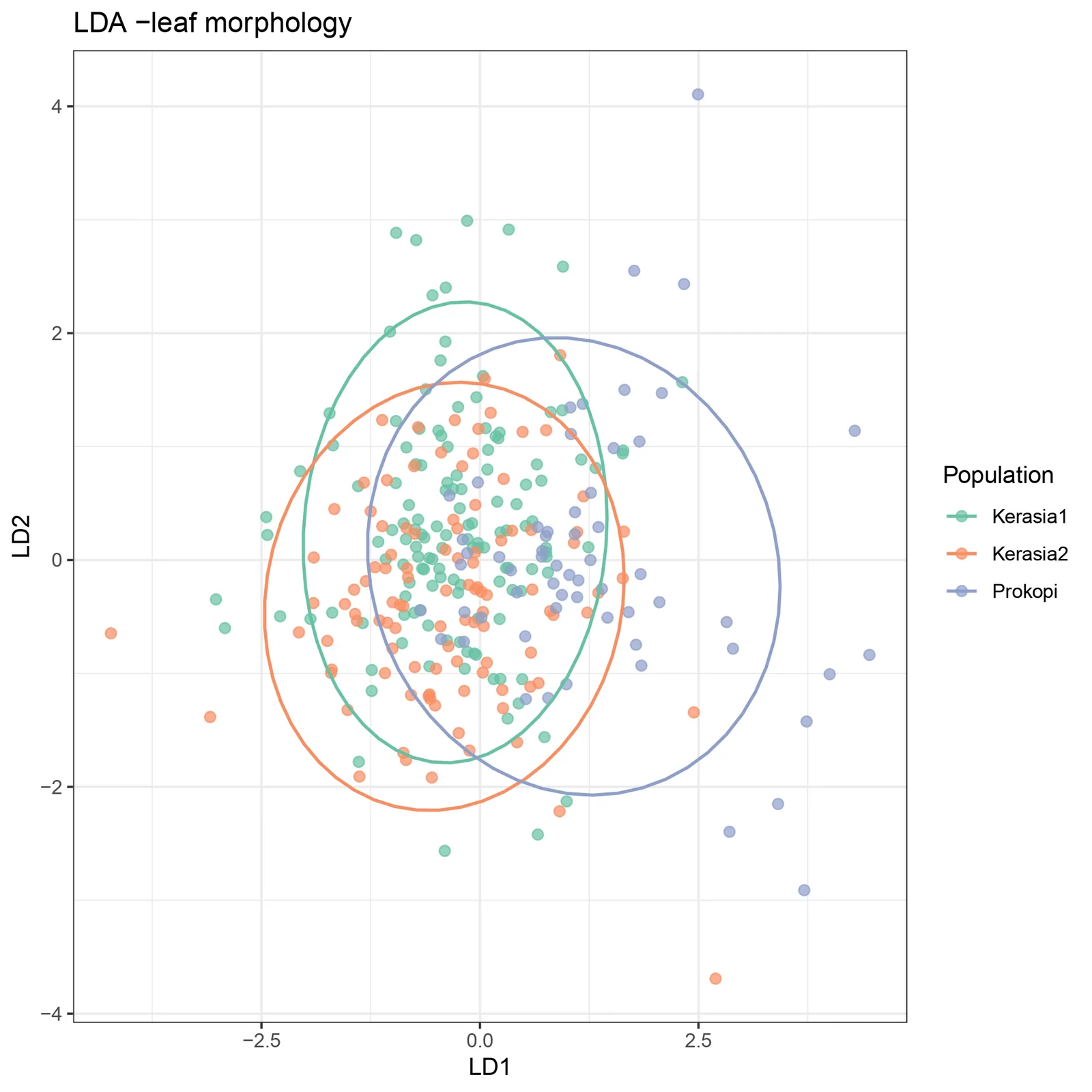
Linear Discriminant Analysis (LDA) plot showing the differentiation amongst the three Q. trojana subsp. euboica populations, based on 14 leaf morphometric variables. Points represent individual leaves, colour-coded by population (Kerasia1, Kerasia2, Prokopi).

**Table 1.** Morphometric parameters and their mathematical definitions used for the characterisation of leaf shape. All linear and area measurements were converted from pixels to physical units (mm and mm^2^), based on individual image calibration (scikit-image and regionprops function through Python v.3.12.x scripts).

**Trait**	**Definition**
Leaf area (mm^2^)	Pixel area of the segmented region
Perimeter (mm)	Contour perimeter
Major axis length (mm)	Length of the major axis of the fitted ellipse
Minor axis length (mm)	Length of the minor axis of the fitted ellipse
Aspect ratio	Major axis / minor axis
Circularity	4 π x area / perimeter^2^
Solidity	Leaf area / convex hull area
Maximum Feret diameter (mm)	Maximum caliper diameter
Minimum Feret diameter (mm)	Minimum caliper diameter
Convex area (mm^2^)	Area of the convex hull
Width-to-length ratio	Minor axis / major axis
Base-to-wide ratio	Distance from leaf base to widest point / major axis length
Petiole-to-lamina ratio	Petiole length / lamina length
Teeth number (n)	Number of marginal teeth, counted manually from scanned images
